# Ramadan Intermittent Fasting and Injury Epidemiology in Mid and Late Adolescent Elite Male Football Players: A Prospective Cohort Study of Incidence, Burden, Training Load, and Psychophysiological Well‐Being

**DOI:** 10.1002/ejsc.70249

**Published:** 2026-09-09

**Authors:** Noureddine Rejab, Fatma Chaari, Mohamed Ali Nabli, Sonia Sahli, Haithem Rebai

**Affiliations:** ^1^ Tunisian Research Laboratory ‘Sports Performance Optimization’ (LR09SEP01) National Center of Medicine and Science in Sports (CNMSS) Tunis Tunisia; ^2^ Research Laboratory, Movement—Interactions, Performance, MIP Le Mans France

**Keywords:** chronobiology disorders, circadian rhythm, hooper index, muscle strain, postural balance, session rating of perceived exertion

## Abstract

This study investigated the influence of Ramadan fasting on injury incidence, training load, subjective well‐being, postural control, and dietary intake in competitive mid‐ and late elite male adolescent football players across three study phases. A prospective observational cohort design was employed over 13 consecutive weeks in 101 elite male youth football players (U17, *n* = 56; U19, *n* = 22; U21, *n* = 23). Three phases were identified: Pre‐Ramadan (Weeks 1–4), Ramadan (Weeks 5–8), and Post‐Ramadan (Weeks 9–13). Weekly session‐RPE training load, Hooper Index, anthropometrics, and 3‐day dietary records were collected prospectively. Injury surveillance was conducted according to the UEFA guidelines. Forty‐six players (45.5%) sustained ≥ 1 time‐loss injury over 10.419.3 h of exposure. Injury incidence increased during Ramadan from 3.76 to 6.83/1.000 h (RR = 1.816, 95% CI: 0.904–3.651; + 81.6%), with injury burden doubling from 55.16 to 111.68 days/1.000 h (+102.5%). Perceived training load (+134.3 AU; *χ* ^2^ = 106.77, *p* < 0.001; *d* = 1.79) and Hooper Index (+1.99 points; *χ* ^2^ = 122.16, *p* < 0.001; *d* = 8.97) increased significantly. Muscular injuries represented 69.6% of Ramadan injuries, while osseous injuries occurred exclusively during Ramadan. Protein intake decreased (26.30%–23.01%; *p* < 0.001; *d* = 3.68), with increased carbohydrate contribution (49.36%–54.13%; *p* < 0.001). Ramadan intermittent fasting was associated with a clinically significant elevation in injury incidence and burden in mid‐ and late elite male adolescent football players, occurring concurrently with increased perceived training load, progressive psychophysiological deterioration, and reduced dietary protein intake. These findings underscore increased injury risk during Ramadan and the need for tailored prevention.

## Highlights

- Ramadan intermittent fasting increased injury incidence by 81.6% and doubled injury burden in elite adolescent male football players compared with the pre‐Ramadan period.
- Ramadan was associated with higher perceived training load, poorer psychophysiological well‐being, and reduced dietary protein intake, indicating compromised recovery during fasting.
- Muscular injuries predominated during Ramadan, while bone injuries occurred exclusively during the fasting period, suggesting phase‐specific changes in injury patterns.
- These findings identify Ramadan as a high‐risk period for youth football and support implementing individualized training, recovery, and nutritional strategies to reduce injury risk.

## 1 Introduction

Football is one of the most widely practiced competitive team sports worldwide, with a substantial proportion of professional and elite youth players competing in regions where Islamic culture and practices are predominant. During the Islamic lunar month of Ramadan, observant athletes undertake total abstention from food, fluid, and oral medication between dawn and sunset (Maughan et al. 2012). Additionally to the intermittent fasting, altered sleep‐wake cycles (Lolli et al. 2025), and constrained recovery opportunities may challenge physiological homeostasis in elite players (Chtourou and Souissi 2012). Surprisingly, sleep duration was not often adversely impacted, although quality and efficiency were decreased during Ramadan (DeLang et al. 2022). These changes lead to potential diurnal fluctuations in neuromuscular and cognitive performance (Chtourou and Souissi 2012; Maughan et al. 2012), and often had a negative influence on physical performance variables (DeLang et al. 2022).

Besides, in athletic populations, chronic sleep restriction has been identified as an independent contributor to musculoskeletal injury risk, with inadequate sleep duration associated with substantially higher injury incidence (approximately 1.7 times) compared with sufficient sleep (Bouzid et al. 2019; Chamari et al. 2012; Chtourou and Souissi 2012). Against this mechanistic background, the injury epidemiology of youth elite male adolescent football players represents a domain of substantial independent concern. Prospective investigations in youth academy cohorts have reported overall injury incidence rates ranging from 2.5 to 9.4 injuries per 1.000 hours of exposure, with meta‐analytic estimates placing injury burden above 96 days lost per 1.000 hours of exposure (Robles‐Palazon et al. 2022; Ruf et al. 2022). Muscular injuries, particularly hamstring and adductor injuries, represent the predominant cause of time‐loss events, with moderate‐to‐severe injuries (8–28 days of absence) accounting for a disproportionate share of the overall injury burden (Monasterio et al. 2023; Ruf et al. 2022; Wik et al. 2021). Besides, the highest incidence and burden of time‐loss injury occurred in mid adolescent male football players (Materne et al. 2021).

Epidemiological evidence specifically addressing Ramadan fasting as a risk modifier for football injuries remains sparse and methodologically heterogeneous. Chamari et al. (2012) provided the first prospective data in this field, reporting significantly elevated non‐contact and training overuse injury rates in fasting professional players relative to both the pre‐Ramadan and post‐Ramadan phases, despite no concurrent increase in imposed training load. Subsequently, Bouzid et al. (2019) demonstrated that perceived fatigue and muscle soreness were elevated at the end of Ramadan in professional players, with impaired perceived recovery persisting even when biochemical markers of cellular damage remained comparable to pre‐Ramadan values. Nevertheless, another study reported no significant effect of Ramadan on injury incidence for Muslim footballers in Qatar (Eirale et al. 2013). Critically, the effects of Ramadan on injury risk remain insufficiently understood, with no studies conducted in a youth cohort that have simultaneously quantified injury epidemiology, perceived training load, subjective well‐being, postural control, and dietary macronutrient intake within an integrated prospective framework across phase‐delineated periods. The extent to which age category modulates the magnitude of Ramadan‐associated physiological deterioration and injury risk has not been examined within a single study design.

The primary aim of this investigation was therefore to determine whether Ramadan intermittent fasting is associated with a significant elevation in injury incidence rate and burden in mid and late elite male adolescent football players competing in the Tunisian First Division. Secondary aims were to characterize phase‐specific changes in perceived training load, subjective well‐being, postural control, and dietary macronutrient intake, and to assess whether these responses differed across three age categories (U17, U19, U21). It was hypothesized that injury incidence, perceived training load, and well‐being impairment would be highest during the Ramadan phase, with only partial restoration during the subsequent Post‐Ramadan period.

## 2 Methods

### 2.1 Study Design

This study employed a prospective longitudinal observational design conducted over 13 consecutive weeks, during the end of the 2024‐2025 season, encompassing three sequentially demarcated phases: Pre‐Ramadan (BR; Weeks 1–4), Ramadan (R; Weeks 5–8), and Post‐Ramadan (AR; Weeks 9–13). The observational framework was selected to preserve ecological validity by monitoring players within their habitual competitive environment, without manipulating training schedules or nutritional protocols (Batterham and Hopkins 2006). Daily data collection included perceived training load quantification, subjective well‐being assessment, systematic injury surveillance, and repeated evaluation of postural control at four standardized timepoints. The exposure periods per phase were as follows: BR (training: 19 h; match: 12–14 h), *R* (training: 20 h; match: 21 h), and AR (training: 19 h; match: 12–14 h).

### 2.2 Participants

A total of 101 highly trained mid‐ and late adolescent football players were recruited from eight clubs competing in the Tunisian First Division and stratified into three age categories: U17 (*n* = 56; age = 17.0 ± 0.5 years; height = 175.8 ± 4.6 cm; body mass = 67.2 ± 5.0 kg; BMI = 21.8 ± 1.9 kg·m^−2^), U19 (*n* = 22; age = 19.0 ± 0.5 years; height = 180.4 ± 4.4 cm; body mass = 70.2 ± 3.0 kg; BMI = 21.6 ± 1.4 kg·m^−2^), and U21 (*n* = 23; age = 21.0 ± 0.5 years; height = 180.3 ± 2.9 cm; body mass = 74.2 ± 4.7 kg; BMI = 22.8 ± 1.7 kg·m^−2^). Inclusion criteria required active participation in a competitive team, a minimum of 3 years of structured club training, completion of at least four weekly training sessions plus one official match per week, and absence of musculoskeletal injury for at least 2 months preceding baseline assessment. Players missing more than 20% of total training sessions or more than two consecutive sessions during the observation period were also excluded to preserve exposure data integrity. The study protocol was conducted in full accordance with the Declaration of Helsinki (Association 2013) and received approval from the institutional ethics committee (IRB code: CEFMSo_0142_2025).

### 2.3 Experimental Procedures

#### 2.3.1 Anthropometric and Body Composition Assessment

Body composition indices were assessed using bioelectrical impedance analysis (BIA) (Tanita TBF‐401; Tanita Corp., Japan) at the four timepoints: BR‐W1, R‐W1, R‐W4, and AR‐W4. All measurements were conducted following a standardized 12 ± 1 h fast. During Ramadan, BIA sessions were scheduled at a standardized interval relative to Iftar to control for acute hydration variation. Standing height was recorded using a calibrated stadiometer (Holtain Ltd., UK) to the nearest 0.1 cm.

#### 2.3.2 Dietary Intake Assessment

Nutritional intake was evaluated monthly across each study phase using 3‐day food records, validated through structured interviews conducted by a qualified dietitian. Macronutrient and energy data were analyzed using NUTRISOFT‐BILNUT software (version 2.01; Paris, France) and the food‐composition tables of the National Institute of Statistics of Tunisia (1978).

#### 2.3.3 Internal Load Monitoring

Session perceived training load was quantified daily using the session Rating of Perceived Exertion method (sRPE) (Foster et al. 2001), in which participants rated perceived exertion on Borg's CR‐10 scale 15–20 min after each session. The perceived training load was computed as the product of session duration (minutes) and RPE score, expressed in arbitrary units (AU). Players had been familiarized with the Borg CR‐10 scale since the start of the competitive season.

#### 2.3.4 Well‐Being Assessment

Subjective well‐being was monitored using the Hooper Index (Hooper and Mackinnon 1995), administered before each training session and match. Participants rated perceived sleep quality, stress, fatigue, and delayed‐onset muscle soreness (DOMS) on a seven‐point Likert scale (1 = very, very good; 7 = very, very bad). The composite Hooper Index was computed as the arithmetic sum of the four sub‐scales (range: 4–28), with higher scores indicating poorer well‐being.

#### 2.3.5 Postural Control Assessment

Postural Control was assessed using the Y‐Balance Test (YBT), a reliable and valid measure of lower‐extremity neuromuscular performance (Plisky et al. 2006). Assessments were conducted at four pre‐specified timepoints: T1 (1 week pre‐Ramadan), T2 (Ramadan Week 1), T3 (Ramadan Week 4), and T4 (2 weeks post‐Ramadan). Reach distances were recorded in the anterior (ANT), posteromedial (PM), and posterolateral (PL) directions for both limbs. Composite reach scores were normalized to leg length using the formula: [(ANT + PM + PL)/(3 × LL)] × 100 (Chaari et al. 2022; Shaffer et al. 2013). Participants performed three familiarization trials followed by three scored attempts per direction, with hands maintained on the hips throughout (Duncan et al. 2017). In accordance with study design, YBT was administered to a subset of 33 players across different categories. This restriction was due to the time‐intensive nature of the test, which made it difficult to assess all 101 athletes within the same timeframe.

#### 2.3.6 Injury Surveillance

Injury surveillance followed the UEFA epidemiological guidelines (Hägglund et al. 2005). An injury was defined as any musculoskeletal complaint sustained during a scheduled training session or match that resulted in absence from the subsequent training session or match (Butler et al. 2013). Injury incidence rate (IIR) and burden were calculated per 1.000 hours of exposure using the equations proposed by (Fuller et al. 2006):

IIR=(∑Injuries/∑Exposurehours)×1000

Burden=(∑Daysabsent/∑Exposurehours)×1000

Injury severity was classified as: very mild (Grade 1, 0–3 days), mild (Grade 2, 4–7 days), moderate (Grade 3, 8–28 days), and severe (Grade 4, > 28 days) (Bahr et al. 2020; Dhahbi et al. 2024). Each injury was independently coded by the medical staff according to anatomical region, tissue type, mechanism (contact vs. non‐contact), and context (training vs. match). Individual exposure time was recorded daily in hours, separately for training and match participation (Raya‐Gonzalez et al. 2020).

### 2.4 Statistical Analysis

Sample size was determined a priori using G*Power (version 3.1.9.6; Heinrich Heine Universität Düsseldorf, Germany), selecting the test family '*F* tests' and the statistical test 'ANOVA: Repeated measures, within‐between interaction,' consistent with the analytic model subsequently applied to the primary continuous outcomes (training load, Hooper Index) across three phases (BR, *R*, AR) and three age categories (U17, U19, U21). The following input parameters were specified: type of power analysis = a priori; number of groups = 3 (age categories); number of measurements = 3 (study phases); effect size *f* = 0.25 (medium), derived from prior Ramadan‐based investigations reporting moderate‐to‐large changes in internal training load and psychophysiological well‐being indices in team‐sport athletes (Boukhris et al. 2019; Chamari et al. 2012); alpha (two‐sided) = 0.05; power (1 − β) = 0.80; correlation among repeated measures *r* = 0.50; and nonsphericity correction *ε* = one (i.e., sphericity assumed, the default and most conservative G*Power setting in the absence of prior pilot covariance data). Under this specification, G*Power computed the required total sample size as *N* = 42 (df1 = 4, df2 = 78, noncentrality parameter *λ* = 10.50, critical *F* = 2.50), corresponding to a minimum of 14 participants per age category. To account for a conservative 10% anticipated attrition, consistent with dropout rates reported in prior prospective Ramadan cohort studies (Chamari et al. 2012), the minimum per‐group requirement was increased to 20 participants, yielding an adjusted total target sample of 60 participants. The final enrolled cohort of 101 players (U17 = 56, U19 = 22, U21 = 23) substantially exceeded this threshold, providing power in excess of 0.80 for the pre‐specified primary and secondary continuous outcomes. All G*Power input and output parameters are reported in full to permit exact reproduction of this calculation (Cohen 2013; Faul et al. 2007).

All analyses were performed in *R* (version 4.3+; *R* (Team 2023) using the packages lme4, lmerTest, glmmTMB, emmeans, and mice. Normality was assessed using the Shapiro–Wilk test applied to all subgroups and the total sample, supplemented by Q‐Q plots. Variance homogeneity was examined using Levene's test. When the assumption of normality was violated for any outcome variable (pre‐specified criterion: Shapiro‐Wilk test, *p* < 0.05), the corresponding parametric analyses were fully replaced by non‐parametric procedures. Specifically, the Friedman test was used for within‐player repeated‐measures comparisons across the three phases, followed by Wilcoxon signed‐rank tests for pairwise post‐hoc comparisons with Bonferroni adjustment (*α* = 0.0167 for the three pairwise comparisons). Between‐category differences within each phase were assessed using the Kruskal–Wallis *H*‐test as a non‐parametric alternative to the between‐subjects fixed effect. Given the hierarchical data structure (players tested within eight teams), two‐level mixed‐effects models were specified for all continuous repeated outcomes, incorporating random intercepts for both player and team. Intra‐class correlation coefficients (ICCs) were estimated from null models prior to covariate adjustment. A negligible clustering effect (ICC < 0.05) combined with a non‐significant likelihood‐ratio test justified the reduction to single‐level models (Snijders and Bosker 2011). For weekly sRPE and Hooper Index outcomes, linear mixed‐effects models were fitted via restricted maximum likelihood, with phase (BR/R/AR), age category (U17/U19/U21), week (continuous), and their interactions as fixed effects; residual covariance structures (first‐order autoregressive [AR(1)], compound symmetry, and unstructured) were selected by Akaike's Information Criterion (AIC). Pairwise contrasts were computed using the emmeans package with Tukey adjustment for omnibus phase and category effects.

Injury count data were analyzed using Poisson mixed‐effects rate models with log‐transformed exposure hours as an offset term. Overdispersion was diagnosed via the Pearson chi‐square to degrees‐of‐freedom ratio; a ratio exceeding 1.5 triggered pre‐specified refitting with a negative‐binomial mixed‐effects model implemented with the glmmTMB package (Brooks et al. 2017). YBT outcomes and body composition variables were analyzed using a mixed‐model repeated‐measures framework with an unstructured covariance matrix; sphericity was assessed with Mauchly's test, and the Greenhouse‐Geisser epsilon correction was applied when violated (DiStefano and Hess 2005).

Familywise error was controlled using the Bonferroni correction for the two co‐primary outcomes (IIR and burden), and the Benjamini‐Hochberg false discovery rate procedure for all secondary outcome families (Belamjahad et al. 2025). Missing data patterns were examined using Little's MCAR test (Little 1988); variables missing at random (MAR) were handled via multiple imputation (mice, *m* = 20, predictive mean matching (Van Buuren and Groothuis‐Oudshoorn 2011), with estimates pooled using Rubin's rules. Missing YBT data were considered structural by design, as assessments were performed only in injured players, and therefore no imputation was applied. Univariate outliers (beyond μ ± 3 SD) and multivariate outliers (Mahalanobis distance, chi‐square critical value, alpha = 0.001) were retained in primary analyses and excluded in pre‐specified sensitivity analyses. Effect sizes are reported as rate ratios with 95% confidence intervals for count outcomes, Cohen's d for pairwise continuous contrasts, and partial eta‐squared (partial η^2^) for fixed effects in mixed models (Cohen 2013). All tests were two‐sided with alpha set at 0.05, and exact *p*‐values are reported throughout in accordance with STROBE and SAMPL reporting guidelines (Lang and Altman 2015; Vandenbroucke et al. 2014).

## 3 Results

### 3.1 Data Validation and Participant Flow

Normality was rejected for all phase‐level outcomes (Shapiro–Wilk *W* < 0.83, all *p* < 0.001), invoking the pre‐specified substitution of parametric mixed‐effects models with Friedman tests and Wilcoxon signed‐rank post‐hoc procedures with Bonferroni correction (*α* = 0.0167 per family of three contrasts).

The following paragraph presents the baseline characteristics of participants. The three age categories differed significantly in standing height (H = 23.14, *p* < 0.001), body mass (H = 31.03, *p* < 0.001), and BMI (H = 7.41, *p* = 0.025). U21 players were the tallest (180.3 ± 2.9 cm) and heaviest (74.2 ± 4.7 kg); U17 players were the lightest (67.2 ± 5.0 kg). BMI was clinically homogeneous across categories (21.78–22.84 kg·m^−2^). Overall, 25 (44.6%) injured players in U17 (injury events, *n* = 30), 11 (50.0%) in U19 (injury events, *n* = 11) and 10 (43.5%) in U21 (injury events, *n* = 11) were observed, allowing for 46 (45.5%) injured players (injury events, *n* = 52) during the 13‐week observation window.

### 3.2 Primary Outcome: Injury Incidence Rate and Burden

**FIGURE 1:**
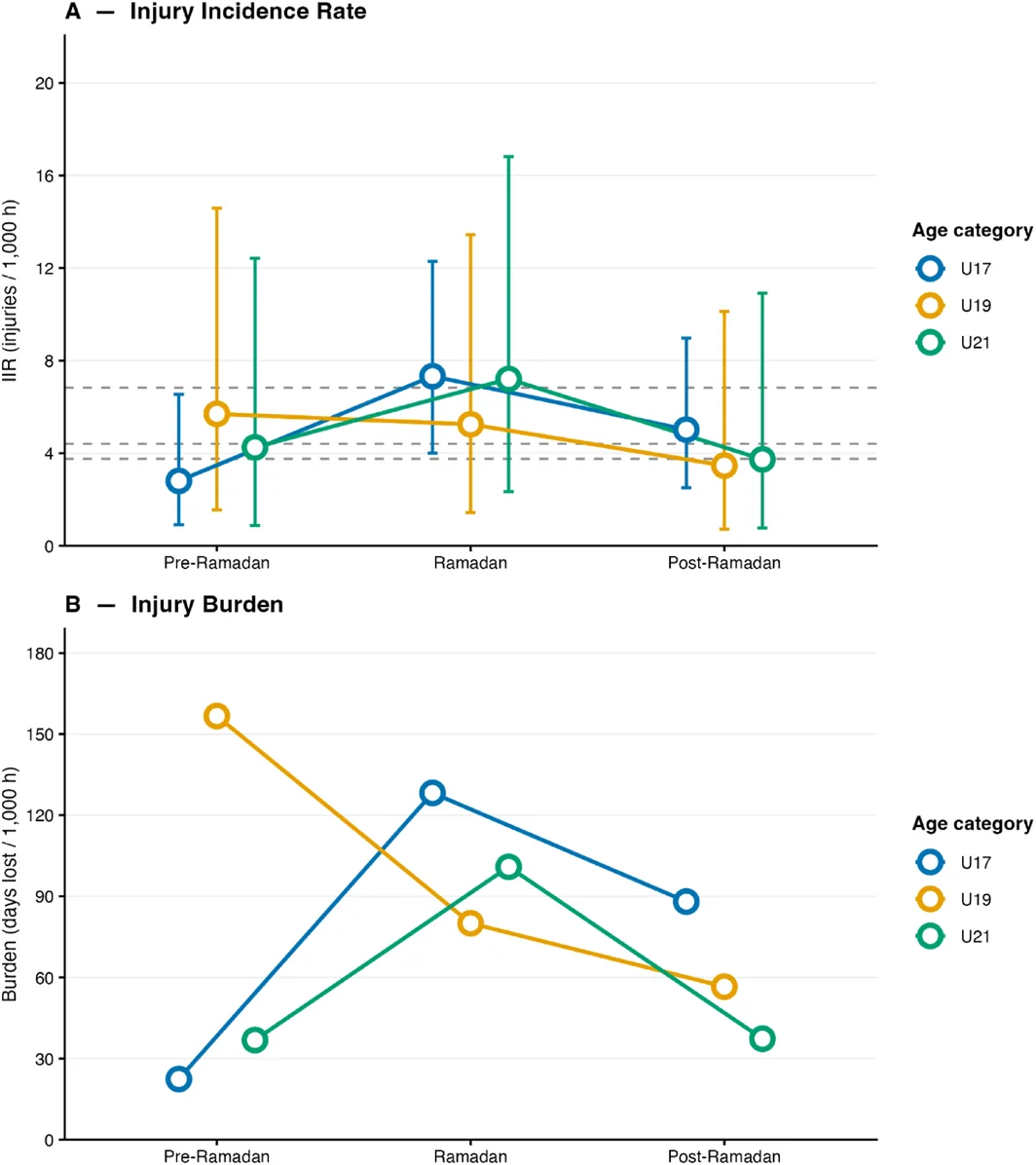
Injury incidence rate and burden across study phases by age category. (A) Injury incidence rate (IIR), expressed as the number of injuries per 1.000 hours of exposure. Error bars represent exact Poisson 95% confidence intervals. Horizontal dashed lines denote the overall cohort mean IIR for each respective phase. (B) Injury burden, expressed as days lost per 1.000 hours of exposure. Data are stratified by age category (U17, U19, U21).

Across the full study period (total exposure = 10.419.3 h; 52 injuries recorded in 46 players), the IIR and burden differed markedly across phases, as displayed in Figure 1. The overall IIR was 3.76/1.000 h (95% CI: 1.94–6.57) during Pre‐Ramadan, rose to 6.83/1.000 h (95% CI: 4.33–10.25) during Ramadan; an 81.6% elevation corresponding to a rate ratio of RR = 1.816 (95% CI: 0.904–3.651); and partially receded to 4.40/1.000 h (95% CI: 2.56–7.05) in Post‐Ramadan (RR vs. Pre‐Ramadan = 1.171, 95% CI: 0.559–2.451). Although both 95% CIs crossed unity, the point estimates and exposure‐weighted trends support a clinically meaningful Ramadan‐associated elevation in injury risk. Injury burden followed a more pronounced trajectory: 55.16 days/1.000 h (Pre‐Ramadan), 111.68 days/1.000 h (Ramadan; +102.5% relative to Pre‐Ramadan), and 70.44 days/1.000 h (Post‐Ramadan). By age category, U17 players exhibited the steepest Ramadan escalation (IIR: 2.80 → 7.33/1.000 h), while U19 showed a numerically declining pattern (5.70 → 5.25 → 3.46/1.000 h).

#### 3.2.1 Between‐Club Variability

Injury incidence rate and burden were compared across the eight participating clubs to assess whether club‐specific training practices could explain the phase effect. Injury incidence rate ranged from 14.71 per 1.000 h (Club 4) to 17.45 per 1.000 h (Club 6), and a chi‐square goodness‐of‐fit test comparing observed and exposure‐weighted expected injury counts showed no significant between‐club difference (*χ* ^2^ = 0.16, *p* = 1.00). Injury prevalence ranged from 30.0% to 62.5% across clubs and also did not differ significantly (*χ* ^2^ = 4.30, df = 7, *p* = 0.75). By contrast, monthly training load (H = 100.0, *p* < 0.001) and Hooper Index (H = 79.32, *p* < 0.001) differed significantly across clubs, indicating expected variation in club‐specific programming rather than a club‐level confound of the Ramadan effect (Table 1).

**TABLE 1:** Club‐level injury incidence, burden, prevalence, exposure, perceived training load, and Hooper Index across the eight participating clubs.

Club	*n*	Exposure (h)	Injuries	Injury prevalence (%)	IIR (/1.000 h)	Burden (days/1.000 h)	TL, BR–R–AR (AU)	Hooper index, BR–R–AR
1 (U17)	12	437.6	7	50.0	16.00	374.77	1605.2–1716.6–1558.0	7.48–9.81–9.87
2 (U17)	17	542.5	8	35.3	14.75	193.57	1426.0–1512.7–1658.6	7.38–9.34–9.26
3 (U17)	14	473.3	7	42.9	14.79	217.61	1400.1–1518.3–1391.6	7.52–9.25–9.52
4 (U17)	13	544.0	8	53.8	14.71	194.85	1403.9–1500.1–1500.5	7.56–9.53–9.33
5 (U19)	12	377.8	6	50.0	15.88	309.70	1270.0–1548.2–1362.5	7.47–9.52–9.83
6 (U19)	10	286.5	5	54.5	17.45	359.51	1308.1–1486.9–1342.8	7.10–9.42–9.34
7 (U21)	13	494.8	8	62.5	16.17	189.99	1324.0–1355.9–1280.8	7.57–9.24–7.55
8 (U21)	10	200.2	3	30.0	14.99	159.88	1327.8–1558.9–1207.6	7.42–9.41–8.37

Abbreviations: AR = Post‐Ramadan; AU = arbitrary units; BR = Pre‐Ramadan; IIR = injury incidence rate; *n* = number of players; *R* = Ramadan; TL = training load (session‐RPE, arbitrary units).

*Note:* Injury incidence rate and burden were compared across clubs using exposure‐weighted descriptive summaries; club‐level heterogeneity in perceived training load and Hooper Index was assessed descriptively and was consistent with differences in club‐specific training practices.

### 3.3 Perceived Training Load and Exertion

**FIGURE 2:**
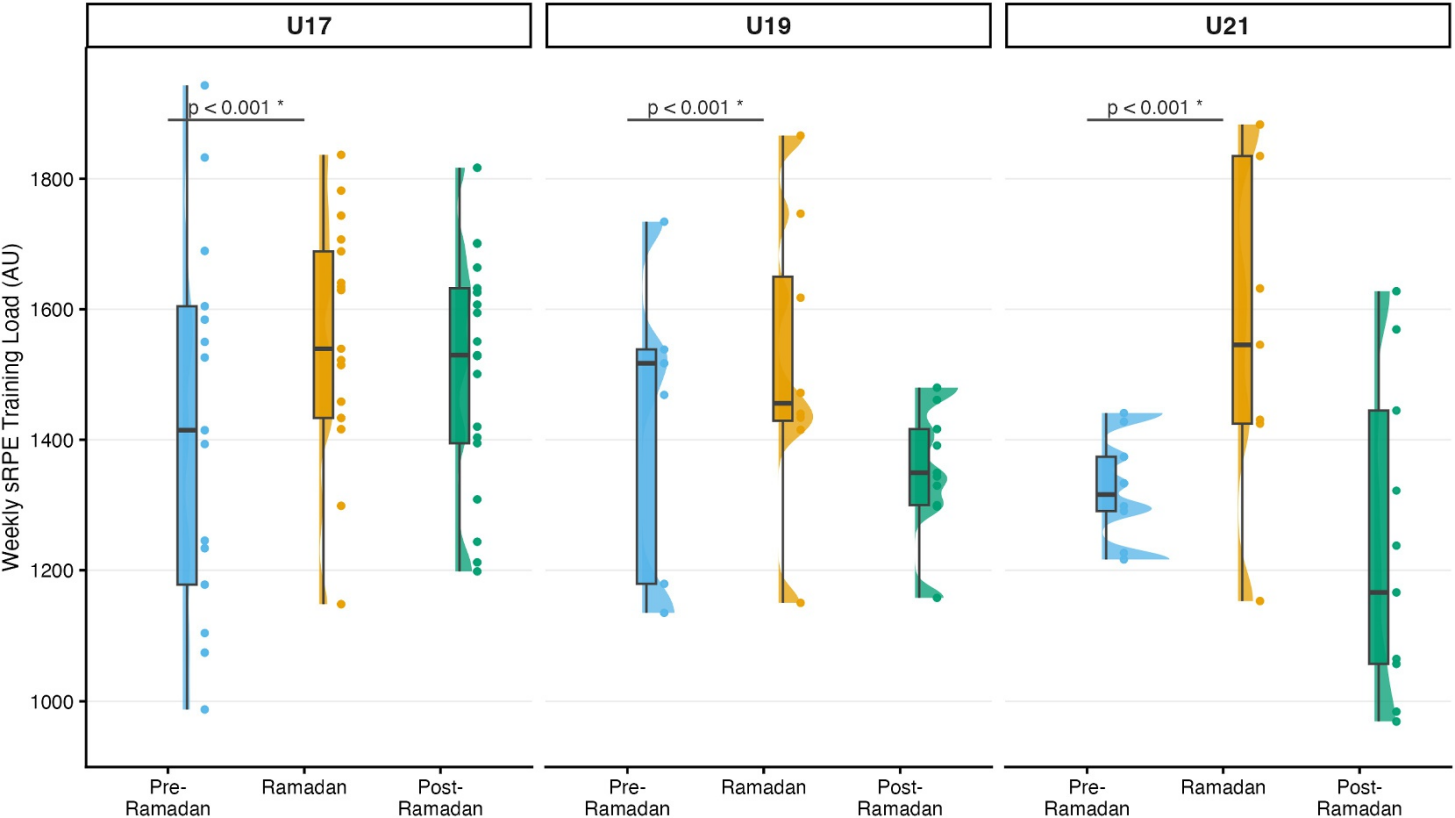
Weekly session‐RPE (sRPE) training load across study phases by age category. Data are presented as raincloud plots comprising half‐violin density distributions, box‐and‐whisker plots indicating the median and interquartile range (IQR), and jittered individual data points representing weekly training loads (AU). Asterisks (*) denote statistically significant differences between phases based on Wilcoxon signed‐rank post‐hoc tests with Bonferroni correction (*p* < 0.001).

**FIGURE 3:**
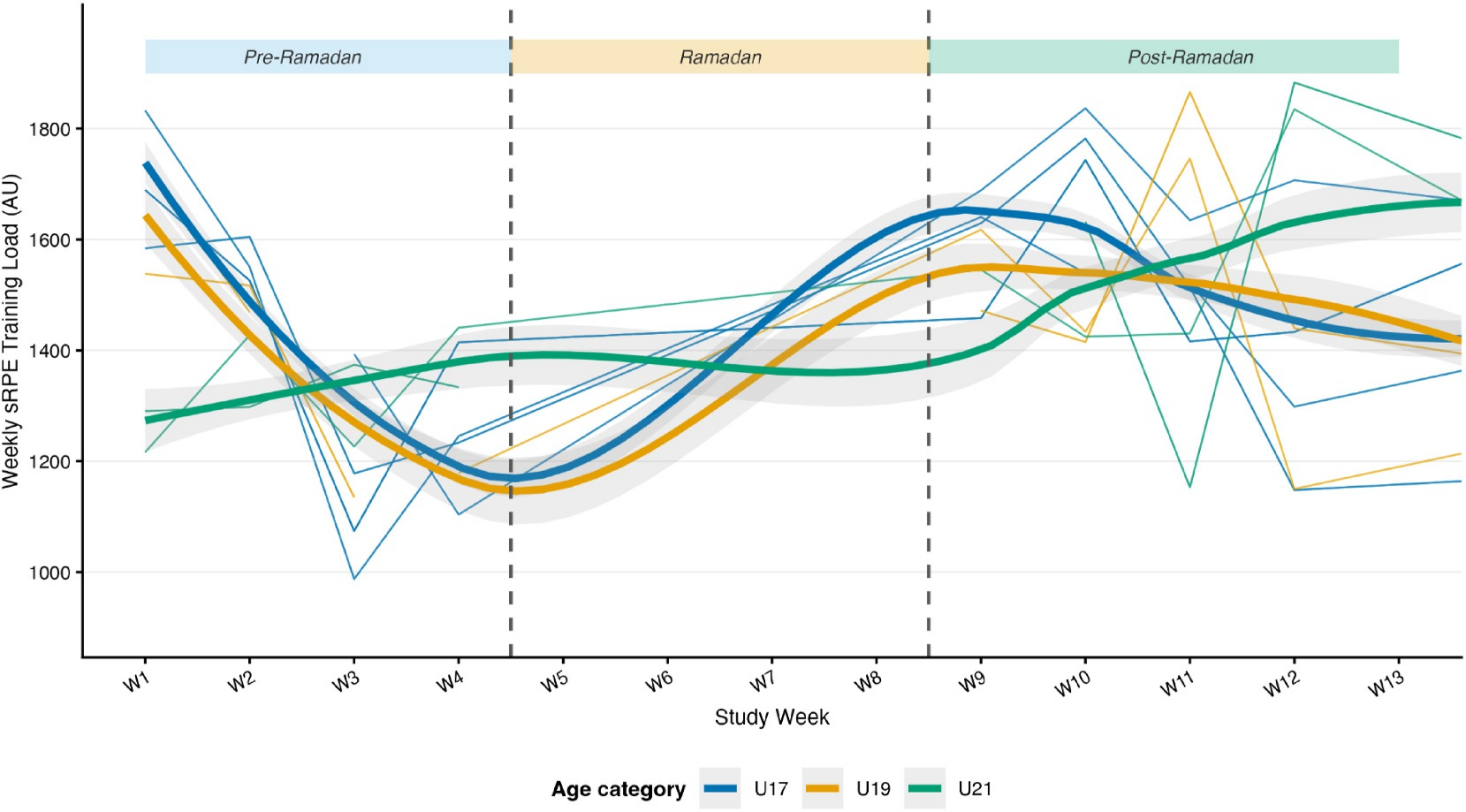
Longitudinal trajectories of individual weekly training load over the 13‐week observation period. Individual player sRPE training loads (AU) are depicted as faint continuous lines (spaghetti plot), color‐coded by age category. Bold, solid lines represent local polynomial regression (LOESS) smoothing curves, with shaded regions indicating the 95% confidence intervals for each age category. Vertical dashed lines demarcate the transitions between the Pre‐Ramadan, Ramadan, and Post‐Ramadan phases.

The distributions of perceived training load and well‐being outcomes across categories are presented in Figure 2 (raincloud plot) and individual longitudinal trajectories in Figure 3 (spaghetti plot).

Weekly session‐RPE training load differed significantly across the three phases (Friedman χ^2^[101] = 106.77, *p* < 0.001). Every one of the 101 players registered a higher Ramadan mean than their Pre‐Ramadan mean (*W* = 0, *p* < 0.001; rank‐biserial *r* = 1.00; Cohen's *d* = 1.79), with a mean increase of +134.3 AU (95% CI: 119.7–149.0). The Ramadan‐to‐Post‐Ramadan transition was also significant (*W* = 1,003, *p* < 0.001; *d* = −0.73; *Δ* = −99.1 AU, 95% CI: −125.7 to −72.5). The Pre‐Ramadan versus Post‐Ramadan contrast did not survive Bonferroni correction (*W* = 1,981, *p* = 0.044), indicating that total load returned close to, but not at, baseline levels. Between‐category load differences were statistically significant in Pre‐Ramadan (H = 82.14, *p* < 0.001) and Post‐Ramadan (H = 82.14, *p* < 0.001), but were attenuated during Ramadan (H = 4.86, *p* = 0.088), reflecting convergence across age groups under fasting conditions. Mean RPE mirrored this pattern (Friedman χ^2^[101] = 39.94, *p* < 0.001): 3.38 ± 0.20 (Pre‐Ramadan), 3.52 ± 0.08 (Ramadan), and 3.54 ± 0.12 (Post‐Ramadan), with all pairwise contrasts significant after Bonferroni correction (Figure 3).

### 3.4 Subjective Well‐Being

The composite Hooper Index deteriorated significantly and progressively from Pre‐Ramadan (7.44 ± 0.14) to Ramadan (9.43 ± 0.17; Δ = +1.99 points, 95% CI: 1.95–2.03; *W* = 0, *p* < 0.001; *d* = 8.97) and remained elevated in Post‐Ramadan (9.20 ± 0.69; *W* = 91 vs. Pre‐Ramadan, *p* < 0.001; *d* = 2.40), with only a modest and marginally significant recovery from the Ramadan peak (*W* = 1,781, *p* = 0.007; *d* = −0.38; Δ = −0.23, 95% CI: −0.35 to −0.11). All four sub‐scales deteriorated significantly from Pre‐Ramadan to both subsequent phases (all Friedman *p* < 0.001, Holm–Šidák corrected). Data highlight that U21 players achieved comparatively greater Post‐Ramadan recovery (HI: 8.17 ± 0.72) than U17 (9.47 ± 0.23) or U19 (9.61 ± 0.25), suggesting age‐related differences in psychophysiological resilience to fasting‐associated fatigue (Table 2).

**TABLE 2:** Internal training load and Hooper Well‐being Index by phase and age category.

Outcome	Phase	U17 (*n* = 56)	U19 (*n* = 22)	U21 (*n* = 23)	Overall (*n* = 101)	Friedman χ^2^	*P*	Post‐hoc summary
Hooper index (4–28)^1^	Pre‐ramadan	7.48 ± 0.08 [7.46–7.50]	7.30 ± 0.19 [7.21–7.38]	7.49 ± 0.10 [7.44–7.53]	7.44 ± 0.14 [7.42–7.47]	122.16	< 0.001	BR versus *R*: *W* = 0, *p* < 0.001, *d* = 8.97^a^ BR versus AR: *W* = 91, *p* < 0.001, *d* = 2.40^a^ *R* versus AR: *W* = 1781, *p* = 0.007, *d* = −0.38^a^
	Ramadan	9.46 ± 0.21 [9.41–9.52]	9.48 ± 0.05 [9.45–9.50]	9.32 ± 0.10 [9.28–9.37]	9.43 ± 0.17 [9.40–9.47]			
	Post‐ramadan	9.47 ± 0.23 [9.41–9.53]	9.61 ± 0.25 [9.50–9.72]	8.17 ± 0.72 [7.86–8.48]	9.20 ± 0.69 [9.07–9.34]			
Sleep (1–7)	Pre‐ramadan	2.04 ± 0.03 [2.04–2.05]	1.97 ± 0.01 [1.97–1.98]	1.95 ± 0.01 [1.95–1.95]	2.01 ± 0.05 [2.00–2.02]	152.61	< 0.001	All pairwise *p* < 0.001^a^
	Ramadan	2.76 ± 0.10 [2.73–2.78]	2.80 ± 0.04 [2.78–2.82]	2.71 ± 0.01 [2.71–2.71]	2.76 ± 0.08 [2.74–2.77]			
	Post‐ramadan	2.81 ± 0.09 [2.78–2.83]	2.79 ± 0.18 [2.71–2.87]	2.58 ± 0.02 [2.57–2.59]	2.75 ± 0.14 [2.72–2.78]			
Stress (1–7)	Pre‐ramadan	1.57 ± 0.03 [1.57–1.58]	1.55 ± 0.06 [1.52–1.58]	1.60 ± 0.01 [1.60–1.61]	1.58 ± 0.04 [1.57–1.58]	164.38	< 0.001	All pairwise *p* < 0.001^a^
	Ramadan	1.88 ± 0.02 [1.87–1.88]	1.84 ± 0.05 [1.82–1.86]	1.80 ± 0.06 [1.78–1.83]	1.85 ± 0.05 [1.84–1.86]			
	Post‐ramadan	1.77 ± 0.02 [1.77–1.78]	1.83 ± 0.01 [1.82–1.83]	1.80 ± 0.04 [1.79–1.82]	1.79 ± 0.03 [1.78–1.80]			
Fatigue (1–7)	Pre‐ramadan	1.99 ± 0.02 [1.99–2.00]	1.94 ± 0.03 [1.92–1.95]	2.01 ± 0.04 [1.99–2.02]	1.98 ± 0.04 [1.98–1.99]	151.62	< 0.001	All pairwise *p* < 0.001^a^
	Ramadan	2.47 ± 0.07 [2.46–2.49]	2.48 ± 0.04 [2.46–2.50]	2.43 ± 0.12 [2.38–2.48]^2^	2.47 ± 0.05 [2.46–2.48]^2^			
	Post‐ramadan	2.48 ± 0.06 [2.46–2.49]	2.54 ± 0.07 [2.51–2.57]	2.35 ± 0.03 [2.34–2.36]	2.46 ± 0.09 [2.45–2.48]			
DOMS (1–7)	Pre‐ramadan	1.87 ± 0.03 [1.86–1.88]	1.84 ± 0.08 [1.80–1.87]	1.93 ± 0.04 [1.92–1.95]	1.88 ± 0.06 [1.87–1.89]	153.68	< 0.001	All pairwise *p* < 0.001^a^
	Ramadan	2.35 ± 0.07 [2.34–2.37]	2.36 ± 0.02 [2.35–2.37]	2.38 ± 0.03 [2.37–2.39]	2.36 ± 0.05 [2.35–2.37]			
	Post‐ramadan	2.41 ± 0.08 [2.39–2.43]	2.45 ± 0.01 [2.45–2.46]	2.26 ± 0.01 [2.26–2.27]	2.39 ± 0.09 [2.37–2.40]			

*Note:* Values: mean ± SD [95% CI] (*n* = 101 per phase). Friedman χ^2^: within‐player repeated measures across three phases. Wilcoxon post‐hoc: Bonferroni‐corrected *α* = 0.0167.

Abbreviations: AU, arbitrary units; DOMS, delayed‐onset muscle soreness; ns, non‐significant.

^a^ Significant after Bonferroni correction (*α* = 0.0167).

^1^Holm–Šidák correction applied across four Hooper sub‐scales. All Friedman χ^2^ significant at *p* < 0.001 unless otherwise stated.

^2^U21 Ramadan Fatigue and Overall Ramadan Fatigue values were derived from the Hooper Index component‐subtraction identity (Fatigue = HI − Sleep − Stress − DOMS) following detection of an irreconcilable data‐entry error in the corresponding source columns of the master dataset; the corrected values are internally consistent with all adjacent sub‐scale entries and with the composite Hooper Index.

### 3.5 Injury Characteristics

The typological and anatomical profile of the 52 recorded injuries is presented in Table 3. Injury type distribution differed significantly across phases (*χ* ^2^ = 18.17, df = 8, *p* = 0.020; Cramér's V = 0.42). Muscular injuries were the dominant type overall (*n* = 28, 53.8%) and were most concentrated during Ramadan (*n* = 16/23, 69.6%); ligamentous injuries predominated in Post‐Ramadan (*n* = 9/17, 52.9%), and osseous injuries occurred exclusively during Ramadan (*n* = 4/23, 17.4%), suggesting a phase‐specific shift in the biomechanical or fatigue‐related underpinning of tissue failure. The mechanism distribution; with non‐contact events accounting for 73.1% of all injuries (*n* = 38); approached but did not reach significance across phases (*χ* ^2^ = 5.24, *p* = 0.073; V = 0.32), though the Ramadan phase exhibited the highest absolute frequency of non‐contact events (*n* = 17/23, 73.9%). Training versus match context did not differ by phase (*χ* ^2^ = 1.27, *p* = 0.529). Anatomically, hamstring (*n* = 10, 19.2%), knee (*n* = 10, 19.2%), ankle (*n* = 9, 17.3%), and adductor (*n* = 7, 13.5%) injuries collectively accounted for 69.2% of all events. Injury severity was uniformly distributed across phases (Kruskal–Wallis H = 2.41, *p* = 0.300), with Grade 3 injuries (8–28 days) predominating (*n* = 30/52, 57.7%), and mean days lost did not differ significantly across periods (H = 1.36, *p* = 0.506). To complement the prevalence‐based injury profile, tissue‐specific injury burden was calculated across the three study periods, which showed higher values during Ramadan specifically for the muscle injury (Table 4).

**TABLE 3:** Injury characteristics by study phase.

Characteristic	Subcategory	Pre‐ramadan (*n* = 12)	Ramadan (*n* = 23)	Post‐ramadan (*n* = 17)	Total (*n* = 52)	Statistic	*p*
Mechanism	Non‐contact	6 (50%)	17 (74%)	15 (88%)	38 (73%)	*χ* ^2^ = 5.24, df = 2, V = 0.32	0.073
	Contact	6 (50%)	6 (26%)	2 (12%)	14 (27%)		
Type	Muscular	6 (50%)	16 (70%)	6 (35%)	28 (54%)	*χ* ^2^ = 18.17, df = 8, V = 0.42	0.020
	Ligament	4 (33%)	3 (13%)	9 (53%)	16 (31%)		
	Bone	0 (0%)	4 (17%)	0 (0%)	4 (8%)		
	Tendon	1 (8%)	0 (0%)	0 (0%)	1 (2%)		
	Other	1 (8%)	0 (0%)	2 (12%)	3 (6%)		
Context	Training	7 (58%)	9 (39%)	7 (41%)	23 (44%)	*χ* ^2^ = 1.27, df = 2	0.529
	Match	5 (42%)	14 (61%)	10 (59%)	29 (56%)		
*Severity* *^a^* *—median [IQR]*		2 [2–3]	3 [2–3]	3 [3–3]	3 [2–3]	KW H = 2.41	0.300

*Note:* Values: *n* (% within phase). χ^2^, Pearson chi‐square; V, Cramér's V; KW, Kruskal–Wallis H. Mean days lost: Pre‐Ramadan 14.7 ± 14.9 d; Ramadan 16.3 ± 9.3 d; Post‐Ramadan 16.0 ± 7.8 d (KW H = 1.36, *p* = 0.506). Top locations (all phases): hamstring (*n* = 10, 19.2%), knee (*n* = 10, 19.2%), ankle (*n* = 9, 17.3%), adductor (*n* = 7, 13.5%).

^a^ Severity: Grade 1 (0–3 d), Grade 2 (4–7 d), Grade 3 (8–28 d), Grade 4 (> 28 d); UEFA classification.

**TABLE 4:** Injury burden by tissue type across study periods.

Phase	Tissue	Injuries, n	Days lost, n	Exposure, h	Burden, days/1000 h	Share of phase burden, %	Kruskal‐wallis H	*p* value
Pre‐ramadan	Muscle	6	66	782.20	84.38	37.50	0.73	0.693
Pre‐ramadan	Ligament	4	52	782.20	66.48	29.55	3.36	0.187
Pre‐ramadan	Other	1	51	782.20	65.20	28.98	1.50	0.221
Pre‐ramadan	Tendon	1	7	782.20	8.95	3.98	NA	NA
Ramadan	Muscle	16	249	1485.20	167.65	66.22	0.73	0.693
Ramadan	Ligament	3	74	1485.20	49.82	19.68	3.36	0.187
Ramadan	Bone	4	53	1485.20	35.69	14.10	NA	NA
Post‐ramadan	Ligament	9	154	1089.16	141.39	56.62	3.36	0.187
Post‐ramadan	Muscle	6	70	1089.16	64.27	25.74	0.73	0.693
Post‐ramadan	Other	2	48	1089.16	44.07	17.65	1.50	0.221

*Note:* Injury burden was calculated as days lost/exposure hours × 1000. Percentages indicate each tissue's contribution to the total burden within each phase. Kruskal‐Wallis *p* values are shown for available cross‐phase comparisons by tissue type. NA = not applicable because no valid cross‐period statistical comparison could be performed for tissue types observed in only one phase.

### 3.6 Postural Control, Body Composition, and Nutritional Intake

The outcomes of the YBT, longitudinal anthropometry, and dietary assessment are consolidated in Table 5.

**TABLE 5:** Y‐balance test, anthropometric, and nutritional parameters across time points.

Domain	Variable	Unit	T1/BR‐W1	*n*	T2/R‐W1	*n*	T3/R‐W4	*n*	T4/AR‐W4	*n*	Friedman χ^2^	*P*
YBT^a^	Composite right	% LL	107.40 ± 14.50 [102.26–112.55]	33	115.34 ± 5.44 [113.38–117.30]	32	115.88 ± 4.97 [113.83–117.93]	25	113.68 ± 4.52 [111.62–115.74]	21	8.54	0.0360
	Composite left	% LL	107.76 ± 14.59 [102.59–112.93]	33	116.38 ± 6.60 [114.01–118.76]	32	116.51 ± 5.14 [114.39–118.63]	25	115.63 ± 4.87 [113.41–117.84]	21	4.32	0.2289
	Asymmetry	%	2.08 ± 1.83 [1.43–2.72]	33	2.63 ± 1.89 [1.95–3.31]	32	2.25 ± 1.92 [1.46–3.04]	25	2.74 ± 1.74 [1.94–3.53]	21	5.30	0.1510
Anthropometrics	Body mass	kg	69.44 ± 5.34 [68.39–70.50]	101	70.95 ± 4.79 [70.00–71.89]	101	69.62 ± 5.62 [68.51–70.74]	101	69.19 ± 4.51 [68.30–70.08]	101	189.59	< 0.001
	BMI	kg·m^−2^	21.99 ± 1.82 [21.63–22.35]	101	22.42 ± 1.67 [22.09–22.75]	101	21.97 ± 1.98 [21.58–22.36]	101	21.68 ± 2.68 [21.15–22.21]	101	183.95	< 0.001
Nutrition^b^	Energy intake	kcal/d	2862.30 ± 93.55 [2843.83–2880.76]	101	2751.45 ± 104.69 [2730.78–2772.11]	101	—	—	3006.98 ± 125.86 [2982.13–3031.83]	101	129.64	< 0.001
	Protein	% Energy	26.30 ± 0.55 [26.19–26.40]	101	23.01 ± 0.60 [22.89–23.12]	101	—	—	24.87 ± 0.63 [24.75–25.00]	101	183.98	< 0.001
	Carbohydrate	% Energy	49.36 ± 0.94 [49.17–49.54]	101	54.13 ± 0.93 [53.95–54.31]	101	—	—	51.77 ± 0.73 [51.62–51.91]	101	197.38	< 0.001
	Fat	% Energy	24.33 ± 1.20 [24.09–24.56]	101	22.81 ± 0.89 [22.63–22.98]	101	—	—	23.57 ± 0.67 [23.44–23.71]	101	166.48	< 0.001

*Note:* Values: mean ± SD [95% CI]; *n* per timepoint listed. Friedman test (within‐subject). YBT assessed in injured sub‐cohort only.

^a^ YBT assessed in injured players only (by design); *n*: *T*1 = 33, *T*2 = 32, *T*3 = 25, *T*4 = 21. Friedman on 21 complete cases (*χ* ^2^ = 8.54, *p* = 0.036 for right composite). Pairwise Wilcoxon *T*1 versus *T*2 (*n* = 32): Right Δ = +8.19 %LL, *W* = 134, *p* = 0.014, *d* = 0.67; Left. Δ = +8.84 %LL, *W* = 99, *p* = 0.002, *d* = 0.73.

^b^ Nutrition assessed monthly by 3‐day food record; T3 (R‐W4) not applicable for dietary data (−). Values are macronutrient % of total energy. Friedman nutrition: Calories *χ* ^2^ = 129.64, *p* < 0.001; Protein *χ* ^2^ = 183.98, *p* < 0.001; Carbohydrate *χ* ^2^ = 197.38, *p* < 0.001; Fat *χ* ^2^ = 166.48, *p* < 0.001. BR‐W1, Pre‐Ramadan Week 1; R‐W1, Ramadan Week 1; R‐W4, Ramadan Week 4; AR‐W4, Post‐Ramadan Week 4; BMI, body mass index.

Concerning YBT, right‐limb composite scores differed significantly across the four timepoints among the sub‐cohort in the complete‐case analysis (*n* = 21; Friedman *χ* ^2^ = 8.54, *p* = 0.036); the left‐limb Friedman test was non‐significant (*χ* ^2^ = 4.32, *p* = 0.229). Maximum‐n pairwise Wilcoxon analysis (*n* = 32) revealed significant bilateral improvements from Pre‐Ramadan baseline (T1) to Ramadan Week 1 (T2): right composite Δ = +8.19%LL (*W* = 134, *p* = 0.014, *d* = 0.67) and left composite Δ = +8.84% LL (*W* = 99, *p* = 0.002, *d* = 0.73). Inter‐limb asymmetry remained within the accepted clinical risk threshold (< 4%) at all timepoints (T1: 2.08 ± 1.83%; T2: 2.63 ± 1.89%; T3: 2.25 ± 1.92%; T4: 2.74 ± 1.74%), with no significant change across phases.

Body mass and BMI both differed significantly across the four measurement timepoints (body mass: Friedman *χ* ^2^ = 189.59, *p* < 0.001; BMI: *χ* ^2^ = 183.95, *p* < 0.001). A transient elevation was observed at Ramadan Week 1 (body mass: 70.95 ± 4.79 kg; BMI: 22.42 ± 1.67 kg·m^−2^) relative to Pre‐Ramadan baseline (69.44 ± 5.34 kg; 21.99 ± 1.82 kg·m^−2^), consistent with pre‐Ramadan dietary loading; values returned to near‐baseline by Post‐Ramadan Week 4 (69.19 ± 4.51 kg; 21.68 ± 2.68 kg·m^−2^), indicating a negligible net body mass change (Δ = −0.25 kg) over the 13‐week observation period.

Nutritional intake parameters differed significantly across all three phases for every macronutrient (all Friedman *p* < 0.001). Total energy intake was lowest during Ramadan (2.751.45 ± 104.69 kcal/d) and highest Post‐Ramadan (3.006.98 ± 125.86 kcal/d), while pre‐Ramadan intake remained intermediate (2862.30 ± 93.55 kcal/d). Ramadan was associated with a significant reduction in protein intake (26.30 ± 0.55% → 23.01 ± 0.60%; *W* = 0, *p* < 0.001; *d* = −3.68) and a reciprocal elevation in carbohydrate intake (49.36 ± 0.94% → 54.13 ± 0.93%; *W* = 0, *p* < 0.001; *d* = +4.03). Following Ramadan, these dietary modifications showed partial recovery toward baseline values, reflecting adaptive nutritional adjustments to altered meal timing and restricted daytime feeding (Table 5).

## 4 Discussion

This study aimed to characterize the phase‐specific effects of Ramadan intermittent fasting on injury incidence rate and burden, perceived training load, subjective well‐being, postural control, and dietary macronutrient intake in 101 elite male youth football players competing across three age categories in the Tunisian First Division.

### 4.1 Injury Incidence and Burden

The primary finding of this investigation is a clinically meaningful elevation in injury incidence rate during Ramadan (6.83/1.000 h; 95% CI: 4.33–10.25) relative to the Pre‐Ramadan phase (3.76/1.000 h; RR = 1.816, 95% CI: 0.904–3.651), accompanied by a doubling of injury burden (111.68 vs. 55.16 days/1.000 h), with the muscle injury has the highest burden (167.65/1000 h). These findings extend the earlier seminal observation by (Chamari et al. 2012), who reported elevated non‐contact (6.8/1.000 h) and overuse training injury rates during Ramadan in professional Tunisian football players despite no significant increase in imposed training load. However, another study found no influence of Ramadan fasting on injury risk in professional first division league of Qatar (Eirale et al. 2013).These conflicting findings may be explained by the presence of coping strategies, such as rescheduling training sessions and matches until after sunset, thereby allowing athletes to rehydrate and replenish their nutritional intake (Eirale et al. 2013). The present study advances the literature by including a larger cohort (*n* = 101 vs. *n* = 42) and adopting a prospective design with systematic weekly exposure recording. Importantly, the increase in injury incidence observed during Ramadan coincided with an increase rather than a decrease in training load. This finding distinguishes the present results from previous contexts in which reduced training load is assumed to mitigate fasting‐related injury risk, thereby providing a novel contribution to the literature on sports epidemiology during Ramadan.

The injury profile further highlighted phase‐specific patterns, with muscular injuries representing 69.6% of all Ramadan‐phase injuries and bone injuries occurring only during this period. Overall, injury type distribution differed significantly between phases. Moreover, the distribution of injury types varied significantly across the different phases (*χ* ^2^ = 18.17, *p* = 0.020; Cramér's V = 0.42). Non‐contact events accounted for 73.9% of Ramadan injuries, a proportion consistent with the fatigue‐mediated neuromuscular failure hypothesis. Chamari et al. (2012) identified a comparable non‐contact predominance during Ramadan and attributed it to the confluence of glycogen depletion, dehydration, and cumulative fatigue. In the present cohort, the fact that hamstring (19.2%), adductor (13.5%), and calf regions collectively accounted for a substantial proportion of all events is consistent with eccentric force‐production vulnerability in fatigued muscles, as described extensively in the football injury literature (Ekstrand et al. 2011). These findings also support previous studies showing that muscle injuries are the most common injury type, with the highest incidence and burden in male football players (Monasterio et al. 2023; Wik et al. 2021).

### 4.2 Perceived Training Load

The concurrent elevation in session‐RPE training load (Δ = +134.3 AU; Cohen's *d* = 1.79; *p* < 0.001) and the progressive deterioration in the composite Hooper Index (Δ = + 1.99 points; *d* = 8.97; *p* < 0.001) provide a plausible mechanistic framework for the observed injury escalation. The convergence of the perceived training load and well‐being data suggests that during Ramadan, players sustained a higher volume of perceived physiological work at a time when their psychophysiological capacity for recovery was compromised. This pattern is consistent with the overreaching model articulated by Halson and Jeukendrup (2004), in which an imbalance between training stress and recovery capacity produces cascading inflammatory, hormonal, and immunological perturbations that elevate tissue vulnerability. Bouzid et al. (2019) demonstrated in professional football players that perceived fatigue and muscle soreness increased during Ramadan even when external training load remained nominally stable, attributing the response to a progressive, sub‐significant accumulation of fatigue that impaired recovery quality. The present data, obtained in a youth population with a substantially larger sample, corroborate and extend that interpretation. Nevertheless, psychosocial factors may also modulate adaptation, as recent findings indicate that religious engagement can positively influence perceived performance and coping during Ramadan (Yacoubi et al. 2026).

### 4.3 Sleep Architecture

Sleep architecture disruption represents a plausible physiological bridge between Ramadan fasting and the observed injury elevation. The Hooper Index sleep sub‐scale deteriorated significantly from Pre‐Ramadan to Ramadan (all pairwise Friedman *p* < 0.001), and this deterioration persisted into the Post‐Ramadan phase. The nocturnal restructuring of eating (Iftar, Suhoor) and augmented social and religious activity during Ramadan systematically compress effective sleep duration and fragment sleep architecture (Bouzid et al. 2019; Chamari et al. 2012; Halson 2008). However, DeLang et al. (2022) showed that sleep duration was not often adversely impacted, although quality and efficiency were decreased during Ramadan. Recent evidence confirms that chronic suboptimal sleep is independently associated with elevated musculoskeletal injury risk in athletes (Bouzouraa et al. 2025; Huang and Ihm 2021). In youth athletes specifically, sleeping fewer than 6–8 hours is associated with injury rates approximately 1.7 times greater than in adequately rested peers (Milewski et al. 2014). Besides, in youth athletes, the onset of Ramadan determined an abrupt reduction in time asleep of ∼ 1 h 30 min in the first period of a night cycle and contributed to additional problems of heterogeneous sleep fragmentation that can influence optimal school learning and performance development processes (Lolli et al. 2025).

### 4.4 Dietary Intake

The nutritional data provide a further, mechanistically independent pathway to elevated injury risk. Total energy intake was lowest during Ramadan (2.751 ± 105 kcal/d), accompanied by a significant reduction in dietary protein (26.30 ± 0.55% to 23.01 ± 0.60%; *d* = 3.68; *p* < 0.001) and a reciprocal carbohydrate elevation. The reduction in protein as a proportion of energy intake is clinically relevant in the context of musculoskeletal tissue maintenance. A systematic review confirms that inadequate protein intake during periods of energetic stress accelerates muscle damage markers and slows repair kinetics (Giraldo‐Vallejo, Cardona‐Guzmán, et al. 2023). The elevated carbohydrate fraction (54.13 ± 0.93% during Ramadan) may reflect compensatory nocturnal hyperphagia focused on rapidly available energy substrates rather than a nutritionally balanced intake pattern (Maughan et al. 2012). In this context, a recent systematic review highlighted the negative effect of Ramadan on nutritional status and fatigue capacities in different sport populations. However, the athletes who maintain their total energy and macronutrient intake, training load, body composition, sleep length and quality are unlikely to suffer any substantial decrements in performance during Ramadan (Ghaffar et al. 2025).

### 4.5 Ramadan Injury and Age Groups

Age‐related variations in injury susceptibility and recovery dynamics need also particular consideration. The U17 players demonstrated the most pronounced increase in injury incidence during Ramadan (IIR: 2.80 to 7.33/1.000 h), while the Hooper Index remained elevated into Post‐Ramadan for both U17 (9.47 ± 0.23) and U19 (9.61 ± 0.25) categories. The U21 players, by contrast, demonstrated comparatively greater Post‐Ramadan recovery (HI: 8.17 ± 0.72). This differential may reflect the greater physiological maturity and accumulated training adaptation of older players, consistent with the premise that experienced athletes possess more refined coping strategies and more efficient recovery responses under nutritional stress (Hottenrott et al. 2022; Monasterio et al. 2023). It may also reflect greater tactical exposure and match time in U17 players, who accounted for the majority of Ramadan injuries (*n* = 14), compounding the fasting‐associated physiological burden. Notably, the U19 category exhibited an atypically elevated Pre‐Ramadan injury burden (156.69 days/1.000 h), substantially exceeding both their own Ramadan burden (80.02 days/1.000 h) and the U17 Ramadan burden (128.22 days/1.000 h). This pattern was driven by four injuries with a mean severity of 27.5 days lost per event, consistent with predominant Grade 3–4 events occurring in the pre‐fasting phase, and likely reflects baseline stochastic variation inherent to small injury counts (*n* = 4) rather than a systematic protective effect of fasting in this subgroup. Furthermore, our findings indicated that muscle injuries were associated with the greatest injury burden. This observation is consistent with a recent study reporting a higher injury burden related to muscle and non‐contact injuries among male youth football players during and after Ramadan compared with the pre‐Ramadan period, in both neuromuscular training and control groups (Belamjahad et al. 2025). Although perceived training load and Hooper Index scores varied across the eight clubs, injury incidence and burden were largely comparable between clubs, suggesting that the Ramadan‐associated injury pattern was not driven by a small number of clubs with atypical injury exposure.

### 4.6 Postural Control

The YBT findings, restricted by design to the players sub‐cohort, revealed bilateral composite score improvements from Pre‐Ramadan to Ramadan Week 1 (right: Δ = +8.19%LL, *d* = 0.67; left: Δ = +8.84%LL, *d* = 0.73) with inter‐limb asymmetry consistently below the established clinical risk threshold (< 4%) at all timepoints. Within this sub‐cohort specifically, postural control as measured by the YBT was not globally impaired at the time of injury assessment, a finding consistent with evidence that YBT composite scores do not universally deteriorate under Ramadan conditions when testing is scheduled appropriately relative to Iftar (Belamjahad et al. 2025). Because the YBT protocol was by design restricted to a limited number of players, these observations cannot be extrapolated to the full cohort of 101 participants.

Some methodological considerations merit acknowledgment. The absence of objective hydration and hormonal biomarkers limits mechanistic specificity. Considering that the cohort was recruited from eight clubs, potential heterogeneity may exist between clubs in terms of injury occurrence and perceived training load. Exploring this aspect would be of interest in future research. External load data (GPS‐derived distance) were not available, precluding differentiation between increases in perceived and mechanical load. Future investigations should adopt a more comprehensive monitoring approach by combining objective sleep assessment (actigraphy), bioelectrical hydration evaluation, external load quantification, and randomized nutritional intervention designs. Such approaches would strengthen causal inference and facilitate the development and validation of targeted, phase‐specific countermeasures.

### 4.7 Practical Recommendations

Strength and conditioning staff should reduce total weekly session‐RPE training load and preserving adequate inter‐match recovery intervals during Ramadan (Rebai et al. 2013). Besides, registered dietitians embedded within club programs should intervene proactively to optimize the nocturnal dietary window, prioritizing protein targets of at least 1.6–2.0 g/kg/day distributed across Iftar and Suhoor meals (Giraldo‐Vallejo, Cardona‐Guzman, et al. 2023). Sleep hygiene protocols, including structured pre‐sleep routines and post‐Iftar nap opportunities, should be integrated into the club's daily Ramadan program to mitigate circadian disruption and its downstream consequences for injury susceptibility (Huang and Ihm 2021).

## 5 Conclusion

This prospective observational cohort study demonstrated that Ramadan intermittent fasting was associated with a clinically significant 81.6% elevation in injury incidence rate and a doubling of injury burden relative to the Pre‐Ramadan phase in 101 mid‐ and late elite male adolescent football players, occurring concurrently with an increase in perceived training load, progressive deterioration in all Hooper Index sub‐scales, and a significant reduction in dietary protein intake. During Ramadan, muscular and bone injuries were the predominant injury types, while non‐contact mechanisms represented almost 75% of all events. These results identify Ramadan as a critical injury‐risk period in competitive mid‐ and late male football and indicate that the elevated injury incidence is not attributable solely to changes in training load.

## Funding

The authors have nothing to report.

## Conflicts of Interest

The authors declare no conflicts of interest.

## Data Availability

The data that support the findings of this study are available on request from the corresponding author. The data are not publicly available due to privacy or ethical restrictions.

## References

[ejsc70249-bib-0001] Association, W. M . 2013. “World Medical Association Declaration of Helsinki: Ethical Principles for Medical Research Involving Human Subjects.” JAMA 310, no. 20: 2191–2194. 10.1001/jama.2013.281053. 24141714

[ejsc70249-bib-0002] Bahr, R. , B. Clarsen , W. Derman , et al. 2020. “International Olympic Committee Consensus Statement: Methods for Recording and Reporting of Epidemiological Data on Injury and Illness in Sport 2020 (Including STROBE Extension for Sport Injury and Illness Surveillance (STROBE‐SIIS)).” British Journal of Sports Medicine 54, no. 7: 372–389. 10.1136/bjsports-2019-101969. 32071062 PMC7146946

[ejsc70249-bib-0003] Batterham, A. M. , and W. G. Hopkins . 2006. “Making Meaningful Inferences About Magnitudes.” International Journal of Sports Physiology and Performance 1, no. 1: 50–57. 10.1123/ijspp.1.1.50. 19114737

[ejsc70249-bib-0004] Belamjahad, A. , C. Tourny , A. C. Hackney , et al. 2025. “Effects of Neuromuscular Training Applied During Ramadan on Physical Fitness and Injury Prevention in Highly‐Trained Male Youth Soccer Players.” Sports Medicine—Open 11, no. 1: 34. 10.1186/s40798-025-00831-y. 40192962 PMC11977061

[ejsc70249-bib-0005] Boukhris, O. , H. Hsouna , L. Chtourou , et al. 2019. “Effect of Ramadan Fasting on Feelings, Dietary Intake, Rating of Perceived Exertion and Repeated High Intensity Short‐Term Maximal Performance.” Chronobiology International 36, no. 1: 1–10. 10.1080/07420528.2018.1513943. 30207750

[ejsc70249-bib-0006] Bouzid, M. A. , A. E. Abaidia , M. Bouchiba , et al. 2019. “Effects of Ramadan Fasting on Recovery Following a Simulated Soccer Match in Professional Soccer Players: A Pilot Study.” Frontiers in Physiology 10: 1480. 10.3389/fphys.2019.01480. 31866876 PMC6909883

[ejsc70249-bib-0007] Bouzouraa, E. , W. Dhahbi , A. Ferchichi , et al. 2025. “Single‐Night Sleep Extension Enhances Morning Physical and Cognitive Performance Across Time of Day in Physically Active University Students: A Randomized Crossover Study.” Life 15, no. 8: 1178. 10.3390/life15081178. 40868825 PMC12387293

[ejsc70249-bib-0008] Brooks, M. E. , K. Kristensen , K. J. Van Benthem , et al. 2017. glmmTMB Balances Speed and Flexibility Among Packages for zero‐inflated Generalized Linear Mixed Modeling.

[ejsc70249-bib-0009] Butler, R. J. , M. E. Lehr , M. L. Fink , K. B. Kiesel , and P. J. Plisky . 2013. “Dynamic Balance Performance and Noncontact Lower Extremity Injury in College Football Players: An Initial Study.” Sports Health 5, no. 5: 417–422. 10.1177/1941738113498703. 24427412 PMC3752196

[ejsc70249-bib-0010] Chaari, F. , S. Boyas , S. Sahli , et al. 2022. “Postural Balance Asymmetry and Subsequent Noncontact Lower Extremity Musculoskeletal Injuries Among Tunisian Soccer Players With Groin Pain: A Prospective Case Control Study.” Gait & Posture 98: 134–140. 10.1016/j.gaitpost.2022.09.004. 36115130

[ejsc70249-bib-0011] Chamari, K. , M. Haddad , D. P. Wong , A. Dellal , and A. Chaouachi . 2012. “Injury Rates in Professional Soccer Players During Ramadan.” Journal of Sports Sciences 30, no. 1: 93–102. 10.1080/02640414.2012.696674. 22697802

[ejsc70249-bib-0012] Chtourou, H. , and N. Souissi . 2012. “The Effect of Training at a Specific Time of Day: A Review.” Journal of Strength & Conditioning Research 26, no. 7: 1984–2005. 10.1519/jsc.0b013e31825770a7. 22531613

[ejsc70249-bib-0013] Cohen, J. 2013. Statistical Power Analysis for the Behavioral Sciences. routledge.

[ejsc70249-bib-0014] DeLang, M. D. , P. A. Salamh , H. Chtourou , H. B. Saad , and K. Chamari . 2022. “The Effects of Ramadan Intermittent Fasting on Football Players and Implications for Domestic Football Leagues Over the next Decade: A Systematic Review.” Sports Medicine 52, no. 3: 585–600. 10.1007/s40279-021-01586-8. 34757593

[ejsc70249-bib-0015] Dhahbi, W. , H. Ben Saad , I. Dergaa , M. Souaifi , and K. Chamari . 2024. “Injury Profiling in Male Police Cadets During Initial Training Phase: A Retrospective Cohort Study.” American Journal of Men's Health 18, no. 6: 15579883241304584. 10.1177/15579883241304584. PMC11626666 39651577

[ejsc70249-bib-0016] DiStefano, C. , and B. Hess . 2005. “Using Confirmatory Factor Analysis for Construct Validation: An Empirical Review.” Journal of Psychoeducational Assessment 23, no. 3: 225–241. 10.1177/073428290502300303.

[ejsc70249-bib-0017] Duncan, M. , E. Eyre , and S. Oxford . 2017. “The Effects of 10‐Week Integrated Neuromuscular Training on Fundamental Movement Skills and Physical Self‐Efficacy in 6‐7‐Year‐Old Children.” Journal of Strength & Conditioning Research 32, no. 12: 1–3356. 10.1519/JSC.0000000000001859. 28346315

[ejsc70249-bib-0018] Eirale, C. , J. L. Tol , A. Farooq , F. A. Smiley , and H. Chalabi . 2013. “Does Ramadan Affect the Risk of Injury in Professional Football.” Clinical Journal of Sport Medicine 23, no. 4: 261–266. 10.1097/JSM.0b013e31828a2bfb. 23528844

[ejsc70249-bib-0019] Ekstrand, J. , M. Hägglund , and M. Waldén . 2011. “Injury Incidence and Injury Patterns in Professional Football: The UEFA Injury Study.” British Journal of Sports Medicine 45, no. 7: 553–558. 10.1136/bjsm.2009.060582. 19553225

[ejsc70249-bib-0020] Faul, F. , E. Erdfelder , A.‐G. Lang , and A. Buchner . 2007. “G* Power 3: A Flexible Statistical Power Analysis Program for the Social, Behavioral, and Biomedical Sciences.” Behavior Research Methods 39, no. 2: 175–191. 10.3758/bf03193146. 17695343

[ejsc70249-bib-0021] Foster, C. , J. A. Florhaug , J. Franklin , et al. 2001. “A New Approach to Monitoring Exercise Training.” Journal of Strength & Conditioning Research 15, no. 1: 109–115. 10.1519/1533-4287(2001)015<0109:anatme>2.0.co;2. 11708692

[ejsc70249-bib-0022] Fuller, C. W. , J. Ekstrand , A. Junge , et al. 2006. “Consensus Statement on Injury Definitions and Data Collection Procedures in Studies of Football (Soccer) Injuries.” British Journal of Sports Medicine 40, no. 3: 193–201. 10.1136/bjsm.2005.025270. 16505073 PMC2491990

[ejsc70249-bib-0023] Ghaffar, T. , F. Ubaldi , F. Valeriani , and V. Romano Spica . 2025. “A Review of the Impact of Intermittent Ramadan Fasting on Wellbeing, Nutrition and Physical Performance in Different Sports.” Clinical Nutrition ESPEN 67: 585–598. 10.1016/j.clnesp.2025.03.052. 40209929

[ejsc70249-bib-0024] Giraldo‐Vallejo, J. E. , M. A. Cardona‐Guzman , E. J. Rodriguez‐Alcivar , et al. 2023. “Nutritional Strategies in the Rehabilitation of Musculoskeletal Injuries in Athletes: A Systematic Integrative Review.” Nutrients 15, no. 4: 819. 10.3390/nu15040819. 36839176 PMC9965375

[ejsc70249-bib-0025] Giraldo‐Vallejo, J. E. , M. Á. Cardona‐Guzmán , E. J. Rodríguez‐Alcivar , et al. 2023. “Nutritional Strategies in the Rehabilitation of Musculoskeletal Injuries in Athletes: A Systematic Integrative Review.” Nutrients 15, no. 4: 819. 10.3390/nu15040819. 36839176 PMC9965375

[ejsc70249-bib-0026] Hägglund, M. , M. Waldén , R. Bahr , and J. Ekstrand . 2005. “Methods for Epidemiological Study of Injuries to Professional Football Players: Developing the UEFA Model.” British Journal of Sports Medicine 39, no. 6: 340–346. 10.1136/bjsm.2005.018267. 15911603 PMC1725241

[ejsc70249-bib-0027] Halson, S. L. 2008. “Nutrition, Sleep and Recovery.” European Journal of Sport Science 8, no. 2: 119–126. 10.1080/17461390801954794.

[ejsc70249-bib-0028] Halson, S. L. , and A. E. Jeukendrup . 2004. “Does Overtraining Exist? An Analysis of Overreaching and Overtraining Research.” Sports Medicine 34, no. 14: 967–981. 10.2165/00007256-200434140-00003. 15571428

[ejsc70249-bib-0029] Hooper, S. L. , and L. T. Mackinnon . 1995. “Monitoring Overtraining in Athletes: Recommendations.” Sports Medicine 20, no. 5: 321–327. 10.2165/00007256-199520050-00003. 8571005

[ejsc70249-bib-0030] Hottenrott, L. , M. Möhle , S. Feichtinger , S. Ketelhut , O. Stoll , and K. Hottenrott . 2022. “Performance and Recovery of Well‐Trained Younger and Older Athletes During Different HIIT Protocols.” Sports 10, no. 1: 9. 10.3390/sports10010009. 35050974 PMC8822894

[ejsc70249-bib-0031] Huang, K. , and J. Ihm . 2021. “Sleep and Injury Risk.” Current Sports Medicine Reports 20, no. 6: 286–290. 10.1249/jsr.0000000000000849. 34099605

[ejsc70249-bib-0032] Lang, T. A. , and D. G. Altman . 2015. “Basic Statistical Reporting for Articles Published in Biomedical Journals: The “Statistical Analyses and Methods in the Published Literature” or the SAMPL Guidelines.” International Journal of Nursing Studies 52, no. 1: 5–9. 10.1016/j.ijnurstu.2014.09.006. 25441757

[ejsc70249-bib-0033] Little, R. J. 1988. “A Test of Missing Completely at Random for Multivariate Data With Missing Values.” Journal of the American Statistical Association 83, no. 404: 1198–1202. 10.1080/01621459.1988.10478722.

[ejsc70249-bib-0034] Lolli, L. , W. Gregson , A. Pulford , T. Kanope , E. Lopez , and V. Di Salvo . 2025. “Immediate Effects of Ramadan on Objective Time Asleep in Male Youth Football Players From the Middle East: An Interrupted Time‐Series Study.” International Journal of Clinical Foot 9, no. 2: 152–162. 10.1080/24733938.2024.2340112. 38753763

[ejsc70249-bib-0035] Materne, O. , K. Chamari , A. Farooq , et al. 2021. “Injury Incidence and Burden in a Youth Elite Football Academy: A Four‐Season Prospective Study of 551 Players Aged From Under 9 to Under 19 Years.” British Journal of Sports Medicine 55, no. 9: 493–500. 10.1136/bjsports-2020-102859. 33199359

[ejsc70249-bib-0036] Maughan, R. J. , Y. Zerguini , H. Chalabi , and J. Dvorak . 2012. “Achieving Optimum Sports Performance During Ramadan: Some Practical Recommendations.” Journal of Sports Science and Medicine 30, no. 1: 109–117. 10.1080/02640414.2012.696205. 22769241

[ejsc70249-bib-0037] Milewski, M. D. , D. L. Skaggs , G. A. Bishop , et al. 2014. “Chronic Lack of Sleep Is Associated With Increased Sports Injuries in Adolescent Athletes.” Journal of Pediatric Orthopaedics 34, no. 2: 129–133. 10.1097/bpo.0000000000000151. 25028798

[ejsc70249-bib-0038] Monasterio, X. , S. M. Gil , I. Bidaurrazaga‐Letona , et al. 2023. “The Burden of Injuries According to Maturity Status and Timing: A Two‐Decade Study With 110 Growth Curves in an Elite Football Academy.” European Journal of Sport Science 23, no. 2: 267–277. 10.1080/17461391.2021.2006316. 34767492

[ejsc70249-bib-0039] Plisky, P. J. , M. J. Rauh , T. W. Kaminski , and F. B. Underwood . 2006. “Star Excursion Balance Test as a Predictor of Lower Extremity Injury in High School Basketball Players.” Journal of Orthopaedic & Sports Physical Therapy 36, no. 12: 911–919. 10.2519/jospt.2006.2244. 17193868

[ejsc70249-bib-0040] Raya‐Gonzalez, J. , F. M. Clemente , M. Beato , and D. Castillo . 2020. “Injury Profile of Male and Female Senior and Youth Handball Players: A Systematic Review.” International Journal of Environmental Research and Public Health 17, no. 11: 3925. 10.3390/ijerph17113925. 32492922 PMC7312653

[ejsc70249-bib-0041] Rebai, H. , H. Chtourou , N. Zarrouk , et al. 2013. “Reducing Resistance Training Volume During Ramadan Improves Muscle Strength and Power in Football Players.” International Journal of Sports Medicine 35, no. 5: 432–437. 10.1055/s-0033-1353216. 24048913

[ejsc70249-bib-0042] Robles‐Palazon, F. J. , A. Lopez‐Valenciano , M. De Ste Croix , et al. 2022. “Epidemiology of Injuries in Male and Female Youth Football Players: A Systematic Review and Meta‐Analysis.” Journal of Sport and Health Science 11, no. 6: 681–695. 10.1016/j.jshs.2021.10.002. 34700052 PMC9729930

[ejsc70249-bib-0043] Ruf, L. , S. Altmann , F. Graf , et al. 2022. “Injury Incidence, Severity, and Burden in Elite Youth Soccer Players—A 3‐Year Prospective Study.” Journal of Science and Medicine in Sport 25, no. 9: 737–742. 10.1016/j.jsams.2022.06.003. 35787346

[ejsc70249-bib-0044] Shaffer, S. W. , D. S. Teyhen , C. L. Lorenson , et al. 2013. “Y‐Balance Test: A Reliability Study Involving Multiple Raters.” Military Medicine 178, no. 11: 1264–1270. 10.7205/MILMED-D-13-00222. 24183777

[ejsc70249-bib-0045] Snijders, T. A. , and R. Bosker . 2011. Multilevel Analysis: An Introduction to Basic and Advanced Multilevel Modeling.

[ejsc70249-bib-0046] Team, R. C. 2023. “A Language and Environment for Statistical Computing.” Computing 1. 10.1890/0012-9658(2002)083[3097:CFHIWS]2.0.CO;2.

[ejsc70249-bib-0047] Van Buuren, S. , and K. Groothuis‐Oudshoorn . 2011. “Mice: Multivariate Imputation by Chained Equations in R.” Journal of Statistical Software 45, no. 3: 1–67. 10.18637/jss.v045.i03.

[ejsc70249-bib-0048] Vandenbroucke, J. P. , E. Von Elm , D. G. Altman , et al. 2014. “Strengthening the Reporting of Observational Studies in Epidemiology (STROBE): Explanation and Elaboration.” International Journal of Surgery 12, no. 12: 1500–1524. 10.1016/j.ijsu.2014.07.014. 25046751

[ejsc70249-bib-0049] Wik, E. H. , L. Lolli , K. Chamari , et al. 2021. “Injury Patterns Differ With Age in Male Youth Football: A Four‐Season Prospective Study of 1111 Time‐Loss Injuries in an Elite National Academy.” British Journal of Sports Medicine 55, no. 14: 794–800. 10.1136/bjsports-2020-103430. 33361134

[ejsc70249-bib-0050] Yacoubi, A. , C. Fitouri , S. Kamal , H. B. Jomaa , I. Latiri , and K. Chamari . 2026. “How Religious Engagement Shapes Performance Perception During Ramadan: Insights From Fasting Tunisian Footballers.” British Medical Bulletin 157, no. 1: ldag004. 10.1093/bmb/ldag004. 41627311

